# Lipid-Polymer Hybrid Nanocarriers for Oral Delivery of Felodipine: Formulation, Characterization and *Ex Vivo* Evaluation

**DOI:** 10.34172/apb.2022.081

**Published:** 2021-09-29

**Authors:** Hayder Kadhim Drais, Ahmed Abbas Hussein

**Affiliations:** ^1^Ministry of Health and Environment, Babil Health Directorate, Babil, Iraq.; ^2^Department of Pharmaceutics, College of Pharmacy, University of Baghdad, Baghdad, Iraq.

**Keywords:** Felodipine, Lipid-polymer hybrid nanocarriers, Microwave-based method

## Abstract

***Purpose:*** Felodipine, is a calcium-channel antagonist used for hypertension and angina pectoris. It is practically insoluble in aqueous media and shows low oral bioavailability (15%-20%). This investigation aims to prepare and characterize oral felodipine lipid-polymer hybrid nanocarriers (LPHNs) to increase solubility and control delivery for increasing bioavailability and enhance patient compliance.

***Methods:*** The newly microwave-based method was prepared with felodipine LPHNs (H1-H35) successfully. The (H1-H35) were subjected to thermodynamic stability experiments. After that, select nine felodipine LPHNs (F1-F9) that have smart physical stability for further optimization of different characterization processes.

***Results:*** The felodipine LPHNs (F4) are considered the most optimized formula. It was characterized by lower particle size (33.3 nm), lower PDI (0.314), high zeta potential (13.6 mV), entrapment efficiency is (81.645% w/w), drug loading is (16.329% w/w), the pH value is 4, excellent percent of light transmittance (95.5%), pseudoplastic rheogram, significantly high (*P* < 0.05) dissolution rate with sustained drug delivery and success *ex-vivo* intestinal permeation attributes. The (F4) subject for further investigations of Fourier transformed infrared spectroscopy (FTIR), atomic force microscopy (AFM), and transmission electron microscopy (TEM). The results of FTIR, AFM, and TEM indicate there is no interaction between the felodipine and excipients and that the particulate system in the nanoscale dispersion system confirms the high stability.

***Conclusion:*** The optimized felodipine LPHNs (F1-F9) formulations were smart formulations for sustained oral delivery of felodipine and that F4 was the most optimized formula according to its characterization processes.

## Introduction

Despite the many discoveries of new medicinal molecules, it still has not risen to the level of drug efficacy capable of saving humans from various diseases, as most of the new drug molecules suffer from the problems of solubility and dissolution parameters. The therapeutic effectiveness of active pharmaceutical ingredients depends mainly on the solubility and dissolution attributes. The solubility is a rate-limiting step for hydrophobic drug absorption from the gastrointestinal tract.^1^ Felodipine, a dihydropyridine type calcium-channel antagonist. Its use to treat hypertension, Prinzmetal’s variant angina, and chronic stable angina pectoris. It is practically insoluble in water and suffers from an extensive hepatic first-pass effect that results in low oral bioavailability (15%-20%).^2^ The global interest in nanotechnology has led to the great development of various interesting sciences, especially the sciences of the medical and pharmaceutical industries. Nanoscience provides many opportunities for creativity and innovation in therapeutic methods to serve various medical specialties. The use of nanoscience through the study of drug carriers contributes to opening broad horizons for scientific research in the field of drug development and the delivery of safe and effective drugs to the patient.^3,4^ The oral route of drug administration remains prevailing where giving the drug orally is very important because of its ease, safety, and high desire for patients. Despite that, it suffers from several obstacles, the most important of which are, the lack of bioavailability due to the lack of drug solubility, lack of permeability to the drug, gut instability, and first-pass effect of the liver.^5^ Therefore, when preparing an oral medication, we must choose a system of administration that can overcome the obstacles present in the human gastrointestinal tract. The conventional oral delivery system has many demerits such as poor patient amenability, increased opportunity of dose missing of a drug with a short half-life for which frequent taking is required and presence of typical peak and valley in the blood concentration/time curve lead to fluctuations in therapeutic agent level this will create adverse effects particularly in therapeutic agent with low therapeutic index.^6^ The more recently, more focus and interest is lipid-polymer hybrid nanocarriers (LPHNs) system which is monolithic type nanocarriers system.^7-9^ The LPHNs is a drug delivery system that protects the encapsulated drug from obstacles associated with oral drug delivery, overcome problems associated with the conventional oral delivery system by providing sustained felodipine delivery. The main constituents of LPHNs are polymer and lipid that reflect properties of polymer nanocarriers and lipid-based nanocarriers.^10^

The presence of polymer makes it more control to release of loaded drug whereas the lipid improves drug solubility, the biological membrane penetration and enhance absorption as well as increase entrapment efficiency of drugs in comparison to polymeric nanocarrier and lipid base nanocarriers alone.^11^ The optimized polymer and lipid concentration in LPHNs creates a fantastic delivery system that has bio-compatibility and bio-degradability properties and less toxic in comparison to other polymeric nanoparticles and lipid base nanocarrier systems.^11^ The hybridization between polymeric nanocarriers and lipid based nanoparticles creates a nanosystem that characterizes mainly by:^12^ furiousness, nanoscale particle size, provide sustained drug delivery, higher drug payload, and high stability in the human circulatory system and during formulation storage.

Numerous methods present in prepare the LPHNs as a nanoparticulate delivery system but related with many demerits mainly more cost, spent high time and stability issue of the final dosage form.^11,12^ The newly microwave-based method is inexpensive, economical, stable, rapidly processing on both small and large scale, and absence of impurities. The radiation of microwaves is a form of electromagnetic non-ionizing that has wavelengths about one meter to one millimeter with frequencies 300 MHz to 300 GHz. The microwaves have three main attributes that facilitate them to be employed in pharmaceutical research and industry which are- reflection by metal substances, it is absorbed by pharmaceutical materials, and able to pass through the plastic, glass, paper, and similar ingredients.^13-18^ This research aims to prepare and characterize oral felodipine LPHNs to increase solubility and control felodipine delivery to improve bioavailability and enhance patient compliance.

## Materials and Methods

###  Materials

The felodipine, lauric acid, polysorbate 80, polysorbate 20, span 80, propylene glycol, and sodium hydroxide were purchased from Nanjing Duly Biotech Co., Ltd. (China). The Labrasol, PEG laurate, and PEG oleate were purchased from Beijing Yibai Biotechnology Co., Ltd. (China). The aniseed oil, argan oil, and cardamom oil were purchased from Hemani International KEPZ (Karachi, Pakistan). The Olibanum oil was purchased from AI-Emad for plant oil products(Iraq). The fenugreek oil was purchased from Bar-sur-Loup Grasse (France). The methanol, ethanol, potassium chloride, potassium dihydrogen phosphate_, _and disodium hydrogen phosphate were purchased from Grin land chemical company (United Kingdom). All other solvents and reagents were of analytical grade.

###  Method 

The microwave-based method is a novel technique that has been used to formulate the LPHNs loaded with felodipine. Under magnetic stirrer device at 1000 rpm for 5 minutes, the felodipine, chitosan polymer, and lauric acid were dissolved in a mixture of cardamom oil and PEG-laurate to prepare the hydrophobic phase to mixed with hydrophilic phase ingredients which are polyoxyethylene (20) sorbitan monooleate, propylene glycol, and distilled water according to the optimized quantities. The mixture of the two phases was put in the microwave instrument, Denka YMO-G30LR-30L model. The applied setting was started by the microwave device for a period 15 second, then under a magnetic stirrer device at 1000 rpm for (2-4 minutes) a solution of colloidal properties of felodipine LPHNs will be obtained. Finally, the felodipine LPHNs formulations were stored in a tightly closed container at 25ºC temperature for a thermodynamic stability study of accelerated stability evaluation. The optimized felodipine LPHNs formulations (F1-F9) use immediately for investigation or lyophilized to fill in hard gelatin capsules for further experimental work. In the lyophilization process (freeze-drying), the samples were first frozen at -20 ºC for 6 h, then enter primary drying process at -45ºC and 0.1 mbar for 18 hours. The remaining of water molecules were removed by secondary drying process at 40ºC and 0.1 mbar for 2 hours, using lactose 10% (w/w) as cryoprotectant.^13-18^

###  Evaluation of physical stability of felodipine LPHNs formulations using thermodynamic stability measurements 

The thermodynamic stability studies were achieved by the following tests.^13,19^

####  Centrifugation assay

 The felodipine LPHNs formulations were centrifuged at the 5000 rpm for 30 minutes and checked physical stability parameters of nanovesicles.

####  Heating-cooling assay

The felodipine LPHNs formulations store at 45ºC and 0^o^C temperature using a refrigerator for not less than two days for each temperature. The heating-cooling test explains the effect of temperature on prepared felodipine LPHNs formulations.

####  Freezing–thawing assay

Under two different temperatures which are -21^o^C and 21ºC, the felodipine LPHNs formulations have been tested for physical stability, using a refrigerator not less than one day for each temperature.

 The felodipine LPHNs formulations that have the maximum physical stability were chosen and used directly for investigation or lyophilized to fill in hard gelatin capsules for further experimental work.

###  Characterization of the felodipine LPHNs formulations (F1-F9)

####  Globule size test

Dynamic light scattering (DLS) is a technique used to determine nanocarriers using Horiba instrument, Ltd. Kyoto, Japan. DLS is based on time-dependent fluctuations (a result of the Brownian motion of the dispersed nanocarriers in LPHNs formulation) in the scattering light intensity when the laser beam is passed through the sample. This technique is highly sensitive and the felodipine LPHNs size were measured in three trials.^20,21^

####  Polydispersity index (PDI) measurement

It is a parameter employed in the measurement of the uniformity of nanovesicles within felodipine LPHNs formulations. It measures by the DLS technique. The higher the polydispersity value refers to the lower uniformity of globules size of the felodipine LPHNs formulations where the experiments achieved in triplicate.^20,21^

####  Zeta potential (ZP) assay

The ZP is an index to predict and control the stability of the colloidal dispersion system. It is measured by the DLS technique. The ZP is a parameter associated with the measure of the surface charge which can develop when any substance is put in a liquid medium.^20,21^ This study was performed in three trials.

####  Entrapment efficiency (EE) and drug loading (DL) 

EE is important evidence concerning drug encapsulation success. The quantity of active pharmaceutical agents loaded in the nanocarriers is a consequence of its dissolution in the hydrophobic phase. The entrapment efficiency was determined through the indirect way by determining non entrapped drug (free felodipine molecules) in the supernatant of felodipine LPHNs formulations after achieving centrifugation technique. It can be indirectly determined by the following equation 1:


(1) EE 00=Total drug amount−free drug amount / Total drug amount×100

DL expressed in percentage (%) is the quantity of active pharmaceutical agents present in the nanocarriers divided by the total quantity of lipid present in the nanosystem. It is measured by equation 2:


(2) DL 00=Total drug amount−free drug amount / Total lipid amount×100

The experiments achieved in triplicate for EE and DL.^22,23^

####  The pH determination

It has a great effect on the solubility of the therapeutic agents, formulation attributes, tolerability, formulation stability, and therapeutic agent activity. All these factors make pH determination is an important parameter in felodipine LPHNs formulations. Also, any pH alteration may indicate chemical reactions that can impair the quality of the final product. The digital pH meter was used to calculate the pH of the felodipine LPHNs formulations. Results were being taken in triplicate.^24-26^

####  Percent of light transmittance assay

The felodipine LPHNs formulations (F1-F9) percent of light transmittance was measured by UV-visible spectrophotometer keeping distilled water as blank at 600 nm. The assays were achieved in triplicate.^27^

####  Viscosity measurement

The felodipine LPHNs formulations (F1-F9) were analyzed using Biobase Meihua Trading Co., Ltd. rotational digital rheometer by employing a spindle number (1) at 25°C. The formulations were exposed to different rotating speed in RPM which are (0.1, 0.3, 0.6,1.5, 3, 6, 12, 30 and 60). The experiments were achieved in three trails.^28^

####  In vitro felodipine release experiment

The drug release analysis was achieved by the combination method of (USP dissolving type I apparatus - dialysis bag technique). The study was performed for all felodipine LPHNs (F1-F9) and compared with drug release from a pure drug suspension. The *in vitro* dissolution study occurs for two dissolution media which are HCl buffer pH 1.2 + 0.3% polysorbate 80 solution and phosphate buffer pH 6.8 + 0.3% polysorbate 80 solutions, where the volume of dissolution medium 900 mL for each assay at 37 ± 0.5ºC with constant stirring at 50 rpm. The drug amount in each of felodipine LPHNs (F1-F9) and pure drug suspension was 5mg of felodipine. Samples were withdrawn at predetermined intervals of time (5, 10, 15, 30, 60 minutes, and 2, 4, 8, 12, 24, 36 hours) and filtered by microfilter paper of 0.45 µm pore size. The withdrawn sample was replaced by the fresh dissolution medium to maintain the quantity of dissolution medium constant during the experiment. The felodipine concentration in each sample was determined spectrophotometrically at 361.5 through a UV spectrophotometer.^28,29^ Dissolution experiments were achieved in triplicate and the results were analyzed using analysis of variance (ANOVA) statistical test at level *P* < 0.05. The kinetics study of the felodipine released from the LPHNs was performed by fitting the resultant data into a different kinetic mathematical equation. The value of regression coefficients (R^2^) will indicate the best fit kinetic model. The diffusion exponent (n) will explain the mechanism of felodipine release.^30,31^

####  Ex vivo intestinal permeation study

*Ex vivo* study was achieved on fasted male sheep weighing about 16 kg using the non-everted sac method.^32-34^ The animal slay and anatomize according to the ethics committee in the university of Baghdad/ college of pharmacy. The small intestine was isolated and carefully removed mesentery matter and washed the small intestine by cold normal saline solution. The small intestine is cut into segments of 5 cm in length and 2 cm in diameter. Insert in each segment ligate from one end one capsule of (felodipine LPHNs F1-F9) and (felodipine suspension) where all capsules contain 5 m of felodipine and add 4.5 g of phosphate buffer pH 6.8 solution and ligate the other end. Then, put the intestinal segments of tightly ligated ends in 900 mL of diffusion liquid which is phosphate buffer pH 7.4 solution + 0.3% polysorbate 80 mg using apparatus 1 rotating basket (Biobase Meihua Trading Co., Ltd.). The samples (5 mL) were taken at precalculated intervals of time (5, 10, 15, 30, 60, 90, 120, 150, 180, 210, 240 minutes) and filtered by microfilter paper of 0.45 µm pore size then measure the felodipine content by UV spectrophotometer where the wavelength is 361.5 nm. After each sample withdrawing, replenished with an equal taken volume of by fresh diffusion liquid immediately. The experiments were achieved in triplicate and the results were analyzed statistically using the ANOVA test at *P*< 0.05. The permeability coefficients were determined using Equation 3.


(3) $$M=P_{eff}\;S\;C_{d}\;t_{res}$$

where, M = quantity of therapeutic agent absorbed; Peff = effective membrane permeability (permeability coefficient); C_d_ = apparent luminal drug concentration (initial concentration or C donor); tres = residence time of drug in GI lumen; S = surface area available for absorption

###  The optimization of the formula

The choice of the optimum formula was accomplished to attain the study purpose and this is achieved according to the best results obtained from felodipine LPHNs (F1-F9) characterizations. The optimum formula exposed for further investigations by FTIR, AFM, and TEM.^19,21^

####  Fourier transformed infrared (FTIR) spectroscopy 

The FTIR is an experiment that explains interactions between therapeutic agents and additives or between additives themselves. The spectrum of FTIR for felodipine, components of LPHNs formulation, and the physical mixture at a ratio (1:1) of felodipine with each LPHNs components which are (cardamom oil, polysorbate 80, propylene glycol, and PEG laurate) and with all components of LPHNs formulation is achieved where the spectrum range was selected from 400 cm^-1^ to 4000 cm^1^. The appearance of additional peaks or disappearance of functional peaks indicate the presence of interaction within felodipine LPHNs formulation.^35^

####  Morphology of nanocarrier surface

*A. Atomic force microscopy (AFM) study:* The morphological properties of felodipine LPHNs formulations were explained by AFM angstrom advanced inc. AA3000 USA. AFM analysis was achieved by applying drops of the felodipine LPHNs formulations onto a glass slide and making it dry then measure. It was scanned at a range of 100 MV/s.^19^

*B. Transmission electron microscopy (TEM):* TEM imaging was employed to define the surface structure of felodipine LPHNs. It was achieved by the LPHNs sample deposition on the 400 mesh copper grids of carbon-coated by employ the negative staining process. In short, the colloidal dispersion system of the felodipine LPHNPS was dropped on the TEM grid and remain for a specific time. At last, the grid was the staining process for the grid was achieved with (2% uranyl acetate) which is a specific negative stain, then dried, and visualized by employ TEM at a voltage of 120 kV (Jeol JEM-1400, Jeol Ltd, Tokyo, Japan).^21,22^

###  Statistical analysis

The quantitative data of the investigation obtained as the average of triplicate samples. Statistical analysis was achieved by the excel program. The ANOVA was applied where the level at (*P* < 0.05) was kept as significant while the level (*P* < 0.05) was kept as not significant.^35^

## Results and Discussion

###  Preparation of felodipine lipid-polymer hybrid nanocarriers

After choosing the formulation components, the felodipine LPHNs (H_1_-H_35_) were prepared successfully using the new microwave-based method. All the prepared felodipine LPHNs passed through physical stability study which is thermodynamic stability analysis. The screening process of felodipine LPHNs Formulations according to the thermodynamic stability studies, the outcomes show that all the felodipine LPHNs formulations (H_1_-H_35_) had a stable pharmaceutical constancy. All the felodipine LPHNs formulations maintain an elegant appearance and color that reflect excellent physical stability. According to the results of thermodynamic stability processes and standards exploited for the selection of various felodipine LPHNs formulations, nine formulas were chosen as in Table 1, which are H1 (F1), H6 (F2), H11 (F3), H16 (F4), H17 (F5), H22 (F6), H27 (F7), H29 (F8) and H34 (F9) for characterization processes.

**Table 1:** Selected felodipine LPHNs formulations for characterization and optimization

**Formulation code**	**Felodipine** **% (w/w)**	**Cardamon oil** **% (w/w)**	**Lauric acid** **%(w/w)**	**Chitosan** **% (w/w)**	**PEG-(400) laurate:Polysorbate** **80: Propylene glycol** **% (w/w)**	**Distilled water** **% (w/w) upto**
F1	1	4	1	0.1	15:7.5:7.5	100
F2	1	4	1	0.1	17.5:8.75:8.75	100
F3	1	8	2	0.2	17.5:8.75:8.75	100
F4	1	4	1	0.2	20:10:10	100
F5	1	8	2	0.25	20:10:10	100
F6	1	12	3	0.35	20:10:10	100
F7	1	4	1	0.2	22.5:11.25:11.25	100
F8	1	8	2	0.35	22.5:11.25:11.25	100
F9	1	12	3	0.4	22.5:11.25:11.25	100

###  Characterization of the prepared felodipine LPHNs (F1-F9) formulations 

 The following tests were utilized to characterize the prepared nanoemulsion systems.

####  Globule size test

Particle size is an important attribute of felodipine LPHNs, which impact encapsulation, stability, efficiency, drug release profile, mucoadhesion, biodistribution, and cellular uptake.^36^ The study of the average size of felodipine LPHNs colloidal dispersion system was determined by z- average using the DLS technique. The outcomes were F1 (87.6 nm); F2 (70.1); F3 (146.2 nm); F4 (33.3 nm); F5 (94.2 nm); F6 (978.4 nm); F7 (49.6 nm); F8 (75.1 nm) F9 (809.4). The results indicate that all optimized felodipine LPHNs formulations (F1-F9) had been distributed in colloidal size and constitute a nano dispersed system. According to the results, the analysis of variance shows rejection of the null hypothesis that state (there is no significant correlation between variables of investigation) and accept the alternative hypothesis that state (there is a significant correlation between variables of investigation), therefore there is a significant correlation between the independent variable and particle size at level (*P* < 0.05).

####  PDI measurement

PDI is the physicochemical property of a dimensionless value that determines the felodipine LPHNs homogeneity. Its value ranges from 0 to 1, and smaller values indicate narrower, more homogenous, and a finer particle size distribution while the uniformity of the particle size in the formulation reduces as the increase in polydispersity value.^37^ The felodipine LPHNs formulations are more homogenous when PDI approach toward zero value. PDI was from (0.314 to 0.798). The outcome of PDI indicated that felodipine LPHNs formulations are a homogenous construct that supports successful, stable, and efficient felodipine LPHNs formulations.^36^ The outcome of the analysis of variance indicated a significant correlation between surfactants: co-surfactant blend, lipid content, and chitosan concentrations as independent variables and PDI at level (*P* < 0.05).

####  ZP assay

The zeta potential is important to attribute use to measuring net charge at the plane of shear of diffuse layer for nanocarriers. It is used to determine particle surface charge which is a parameter of the physical stability of a nanodisperse system. The outcome of the mean zeta potential scale was (0.4 to 21 mV). Zeta potential absolute values according to the thumb rule are: the 5 mV show fast aggregation, about 20 mV supply only short-term stability, above 30 mV offer good stability and above 60 mV excellent stability.^38,39^

Theoretically, the system of nanocarriers should have a higher electrical charge on the particle surface to avoid aggregation during the collision in the solutions. This rule applies to nanocarrier systems that depend on DLVO forces to evaluate the physical stability of the nanodisperse system. For felodipine LPHNs formulation, depend on non DLVO forces which are hydration forces and steric forces to achieve physical stability therefore, despite the low value of zeta potential of LPHNs formulations (F1-F9), it still has high stability due to the process of stabilization achieved by non DLVO forces.^40,41^ On the other hand, the thumb rule applies for electric stabilization of small molecular weight particles, but not for large molecular weight particles such as polysorbate 80 and PEG laurate which are nonionic stabilizers that present in felodipine LPHNs.^41^ There is a significant relationship between independent variables and zeta potential at level (*P* < 0.05).

####  Entrapment efficiency (EE) and drug loading (DL) 

The entrapment efficiency and felodipine loading is an important parameter that is used in optimization of felodipine LPHNs formulation. The outcomes of EE (%w/w) were F1 (82.911%); F2 (82.278%); F3 (85.443%); F4 (81.645%); F5 (84.81 %); F6 (86.708 %); F7 (80.379%); F8 (84.177 %) and F9 (86.075%). On the other hand, the results of drug loading (%w/w) were F1 (16.582%); F2 (16.456%); F3 (8.544%); F4 (16.329%); F5 (8.481 %); F6 (5.78 %); F7 (16.075%); F8 (8.418 %) and F9 (5.738%). The results show that all felodipine LPHNs formulations (F1-F9) had fantastic entrapment efficiency and felodipine loading attributes.^22^ The analysis of variance show rejection of null hypothesis that state accept the alternative hypothesis that state therefore there is a significant relationship between independent variables and entrapment efficiency and felodipine loading (dependent variable) at level (*P* < 0.05).

####  The pH determination

The outcome indicates that the felodipine LPHNs formulations(F1-F9) had a suitable acidic pH value in the range of (4–4.4) that is better for oral administration.^42^ It was found that as lipid content increase there is a slight increase in pH due to increase in cardamom oil that contains mainly alpha-terpinyl acetate which is extremely weak basic that neutralize the acidity of lauric acid within the formula.^42^ The analysis of variance shows a significant relationship (*P* < 0.05) between independent variables and pH of formulations.

####  Percent of light transmittance assay

The percent of light transmittance of the felodipine LPHNs formulations (F1-F9) was measured at (600 nm) which is the visible spectrum region where the blank was distilled water. The percent of light transmittance was found in a range (from 90.4% to 97.1%). The outcomes indicate that all the preparations were nearly transparent and felodipine LPHNs formulations (F1-F9) gave a feature of colloidal dispersion.^43,44^ The analysis of variance indicated a significant relationship between independent variables and percent of light transmittance of formulations (dependent variables) at a level (*P* < 0.05).

####  Viscosity measurement

**Figure 1:**
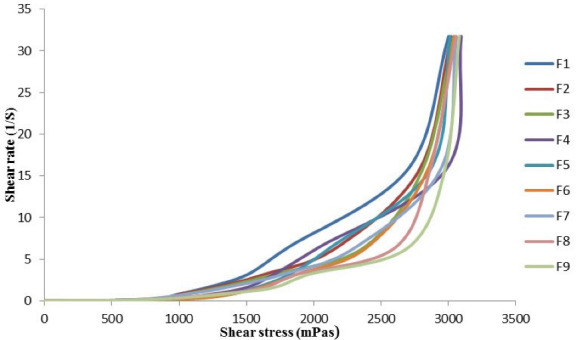
Shear stress against a shear rate of felodipine LPHNs formulations (F1-F9)

The viscosity experiment was achieved successfully using various rotating speeds and different data were obtained which are shear rate, shear stress, and viscosity of felodipine LPHNs. When the plot of shear rate against shear stress, a rheogram chart was achieved as shown in Figure 1. All felodipine LPHNs formulations show non-Newtonian pseudoplastic flow due to it deforms and spouts immediately with applied stress which means it is the shear-thinning feature. The pseudoplastic rheological behavior enhances the stability of felodipine LPHNs formulations due to the decrease rate of aggregation and settling of nanocarriers during long term storage also provide dosage uniformity of therapeutic agents.

####  In vitro felodipine release experiment

The experiment was done using the combinational method which is (USP type I (Basket) - dialysis bag technique in two dissolution media which are HCl buffer pH 1.2 + 0.3 % polysorbate 80 solution and phosphate buffer pH 6.8 + 0.3 % polysorbate 80 solutions. According to the experimental data, there is no bust release from all felodipine LPHNs formulations after instant inundation in the dissolution medium. There was a sustained release process over 36 hours from all optimized felodipine LPHNs formulations (F1-F9).^45^

**Figure 2:**
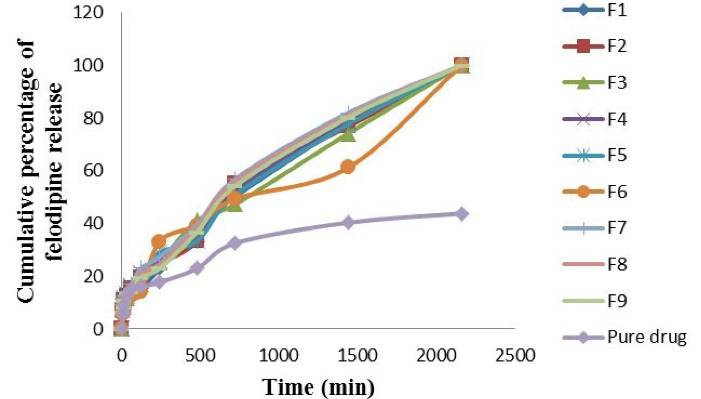
In vitro felodipine release profile from LPHNs formulations (F1-F9) and the pure drug suspension at HCl buffer pH 1.2 + 0.3 % polysorbate 80 solution

In dissolution medium of HCl buffer pH 1.2 + 0.3 % polysorbate 80 solution, the felodipine release profile was significantly higher (*P* value < 0.05) in dissolution rate for F7 and was significantly lower (*P* value < 0.05) in dissolution rate of pure drug as shown in Figure 2. The comparability profile of the felodipine release from optimized LPHNs formulation (F1-F9) and the pure drug suspension explain in the following descending order: F7 > F8 > F9 > F4 > F1 > F5 > F2 > F3 > F6 > pure drug.

**Figure 3:**
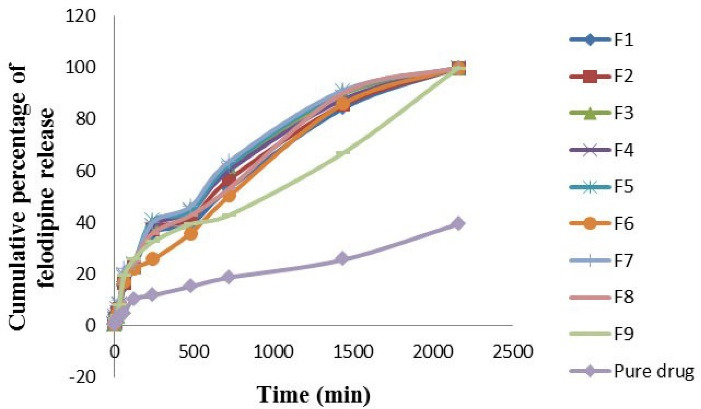
In vitro felodipine release profile from LPHNs formulations (F1-F9) and the pure drug suspension at phosphate buffer pH 6.8 + 0.3 % polysorbate 80 solution

In the dissolution medium of phosphate buffer pH 6.8 + 0.3 % polysorbate 80 solution, the felodipine release profile was significantly higher (*P* value < 0.05) in dissolution rate for F7 and was significantly lower (*P* value < 0.05) in dissolution rate of pure drug suspension as shown in Figure 3. The comparability profile of the felodipine release from optimized LPHNs formulation (F1-F9) and the pure drug suspension explain in the following descending order: F7 > F5 > F8 > F3 > F4 > F6 > F2 > F1 > F9 > pure drug suspension. It was observed that the pure drug suspension gives a lower dissolution rate of felodipine profile in comparison to all optimized LPHNs formulations (F1-F9) because that LPHNs formulations (F1-F9) provide nanoscale particles and have a large surface area that exposes to the dissolution medium and this will allow a higher interaction with the dissolution medium that increases dissolution rate.^46,47^ The significant difference in release profile of felodipine from various optimized LPHNs formulation (F1-F9) and the pure drug suspension indicate there is a significant correlation (*P* value < 0.05) between the PEG laurate, polysorbate 80, and propylene glycol: lipid content: chitosan polymer on the rate of felodipine release.

####  Kinetic analysis of released drug 

The data of in vitro release were applied to various kinetic models to explain the felodipine release mechanism from LPHNs. The release kinetic equations and models that were used were zero-order, first-order kinetic, Higuchi model, and Korsmeyer-Peppas model. The obtained kinetic data of optimized felodipine LPHNs were summarized in Tables 2 and 3. The data analysis explained that an excellent regression coefficient (R^2^) was achieved for Higuchi’s model, which suggested that the release of a drug in the idealistic matrix system in all LPHNs preparations was Higuchi’s diffusion_._ The ‘n’ values (release exponent), of all felodipine LPHNs formulations were significantly higher (*P* < 0.05) than 0.43 indicating that the release of felodipine from LPHNs formulations following non Fickian/anomalous release (diffusion and erosion).^30^

**Table 2:** Correlation coefficient (R^2^) and release exponent (n) of different kinetic models of felodipine LPHNs (F1-F9) and the pure drug released in HCl buffer pH 1.2 + 0.3 % polysorbate 80 solutions

**Formulation code**	**Zero order model** **(R** ^2^ **)**	**First order model** **(R** ^2^ **)**	**Higuchi model** **(R** ^2^ **)**	**Korsmeyer-Peppas model** **(R** ^2^ **)(n)**	
F1	0.97	0.874	0.9826	0.943	0.498
F2	0.965	0.882	0.978	0.932	0.49
F3	0.971	0.866	0.982	0.948	0.515
F4	0.967	0.888	0.981	0.920	0.484
F5	0.968	0.8821	0.979	0.934	0.491
F6	0.942	0.810	0.965	0.952	0.505
F7	0.953	0.912	0.987	0.92	0.487
F8	0.958	0.906	0.984	0.919	0.489
F9	0.964	0.901	0.982	0.942	0.498
Pure drug suspension	0.843	0.89	0.965	0.908	0.413

**Table 3:** Correlation coefficient (R^2^) and release exponent (n) of different kinetic models of felodipine LPHNs (F1-F9) and the pure drug released in phosphate buffer pH 6.8 + 0.3 % polysorbate 80 solutions

**Formulation code**	**Zero order model** **(R** ^2^ **)**	**First order model** **(R** ^2^ **)**	**Higuchi model** **(R** ^2^ **)**	**Korsmeyer-Peppas model** **(R** ^2^ **)(n)**	
F1	0.928	0.927	0.991	0.925	0.74
F2	0.917	0.938	0.992	0.986	0.69
F3	0.895	0.965	0.989	0.924	0.772
F4	0.9044	0.9535	0.992	0.9811	0.664
F5	0.891	0.968	0.988	0.964	0.712
F6	0.951	0.931	0.983	0.981	0.633
F7	0.89	0.972	0.989	0.9819	0.644
F8	0.917	0.9563	0.9884	0.9794	0.658
F9	0.9318	0.8284	0.9706	0.956	0.67
Pure drug suspension	0.939	0.958	0.9755	0.9318	0.479

###  Ex vivo intestinal permeation study

**Figure 4:**
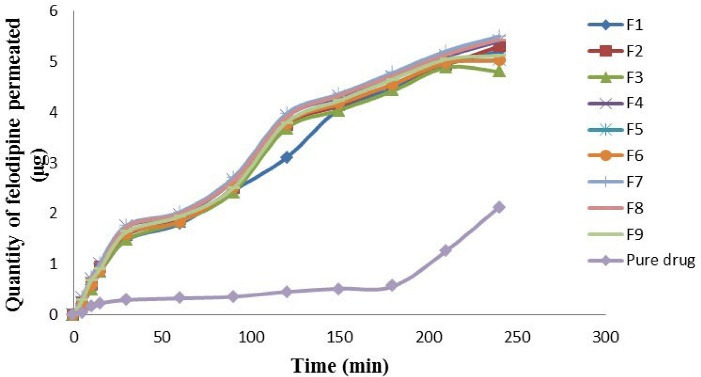
Permeation of felodipine from LPHNs formulations (F1-F9) and pure drug suspension through non-everted sheep intestine

The outcomes of the *ex vivo* intestinal permeation experiment indicate that the permeability coefficient (cm/min) of felodipine was significantly higher (*P* value < 0.05) for F7 and was significantly lower (*P* value < 0.05) for pure drug suspension. The permeability coefficient (cm/min) was calculated after obtaining felodipine flux (μg/mL) as shown in Table 4. The comparability profile of the felodipine LPHNs formulations (F1-F9) and the pure drug is explained in the following descending order: F7 > F8 > F4 > F2 > F9 > F1 > F6 > F5 > F3 > pure drug suspension as shown in Figure 4. It was observed that the pure drug suspension gives a lower permeation coefficient in comparison to all optimized felodipine LPHNs formulations (F1-F9) because that felodipine LPHNs formulations belong to lipid base nanosystem that increases solubility and intestinal permeation also, nanoscale of these hybrid carriers provide large surface area for molecular felodipine libration and rapid absorption to the systemic circulation.^32-34^ The analysis of variance indicates a significant (*P* value < 0.05) correlation between the blend of PEG laurate: polysorbate 80: propylene glycol contents, lipid content, and chitosan polymer and ex vivo intestinal permeation parameter.

**Table 4:** Slope and permeation coefficient for felodipine from LPHNs formulations (F1-F9) and pure drug suspension through non-everted sheep intestine

**Formulation code**	**Flux (μg/mL)**	**Permeability coefficient (cm/min)**
F1	0.0218	0.000000578
F2	0.022	0.000000583
F3	0.0211	0.000000559
F4	0.0223	0.000000591
F5	0.0213	0.000000564
F6	0.0217	0.000000575
F7	0.0227	0.000000601
F8	0.0226	0.000000599
F9	0.0219	0.00000058
Pure drug suspension	0.006	0.000000159

###  The optimization of the formula

The selection of the best-characterized formula of felodipine LPHNs is done according to the desirability process which matches the directions of scientific research to achieve the best control on felodipine delivery. From characterization experiments of felodipine LPHNs formulations (F1-F9) as shown in Table 5, it was found that F4 is more preferred as the best formula that characterized by lower particle size (33.3 nm), lower PDI (0.314), zeta potential (13.6 mV), entrapment efficiency is (81.645 %w/w), drug loading is (16.329 % w/w), the pH value is (4), excellent percent of light transmittance (95.5%), significantly high (*P* < 0.05) dissolution rate and fantastic ex vivo intestinal permeation attributes. The selected formula (F4) is subject to further investigations of FTIR, AFM, and TEM.

**Table 5:** Summary of characterization results of felodipine LPHNs formulations (F1-F9)

**Formulation Code**	Globule size (nm)*	PDI*	Zeta potential*	Entrapment efficiency % (w/w)*	**Drug loading** % (w/w)*	pH*	Percent of light transmittance*
F1	87.6	0.538	6.4	82.911	16.582	4.1	97.1
F2	70.1	0.401	3.3	82.278	16.456	4.1	96.3
F3	146.2	0.547	2.3	85.443	8.544	4.2	94.3
F4	33.3	0.314	13.6	81.645	16.329	4	90.5
F5	94.2	0.54	10.3	84.81	8.481	4.1	95.2
F6	978.4	0.729	3.1	86.708	5.78	4.2	92.4
F7	49.6	0.374	21.1	80.379	16.075	4.1	90.4
F8	75.1	0.335	10.2	84.177	8.418	4.3	92.3
F9	809.4	0.798	0.4	86.075	5.738	4.4	95.5

*Values are expressed as mean ± SD (n = 3).

####  FTIR spectroscopy 

**Figure 5:**
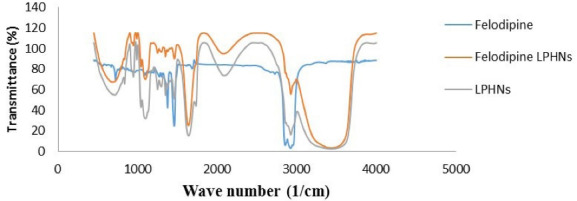
FTIR spectra of pure felodipine, LPHNs, and physical mixture (1:1) of felodipine: LPHNs

The analyzed pure felodipine peaks were 2870, 2960 (Methyl C-H asym./sym. stretch), 1375 (Methyl C-H asym./sym. bend), 720 (Skeletal C-C vibrations), 1490 (Aromatic ring stretch), 2975 (Aromatic C-H stretch), 1050 (Aromatic C-H in-plane bend), 890 (Aromatic C-H out-of-plane bend), 740 (1,2-Disubstitution, ortho), 3480 (Heterocyclic amine, NH stretch) and 1608 (Carboxylate). These characteristic peaks indicate the purity of felodipine. The FTIR spectrum of LPHNs components is (lauric acid, cardamom oil, chitosan polymer, PEG laurate, polysorbate, and propylene glycol) was also studied the spectrum of a physical mixture (1:1) of felodipine with each LPHNs component as shown in Figure 5. The results show that the characteristic peaks of felodipine not affected and prominently observed in all described IR spectra indicating there is no incompatibility and no interaction between the felodipine and excipients.^28^

####  Morphology of nanocarrier surface

**Figure 6:**
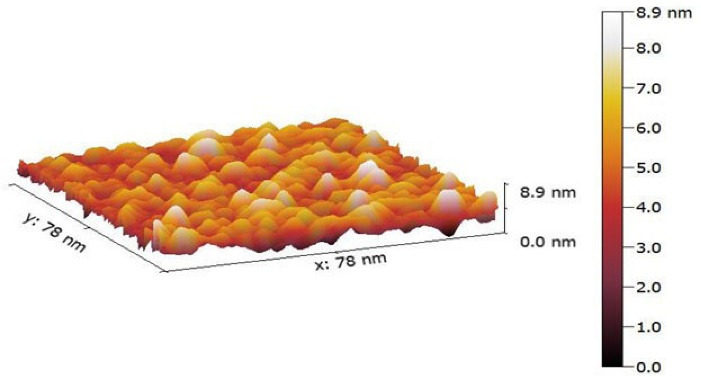
AFM 3D image of felodipine LPHNs (F4) where scanning area is 78 nm * 78 nm

*A. AFM study:* The outcomes indicate that the morphology of optimized felodipine LPHNs (F4) was a nearly globular shape with a smooth surface with the rate of particle size (10 nm -25 nm) as shown in Figure 6. The investigated formulation (F4) does not show particle aggregation and this indicates the stability of felodipine LPHNs (F4).^19^

**Figure 7:**
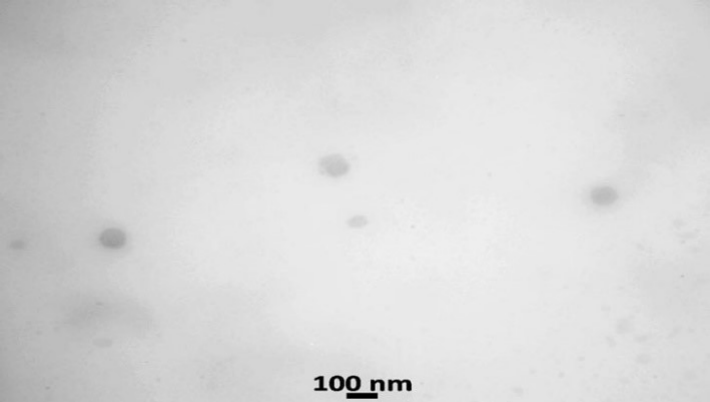
Transmission electron microscopic image of felodipine LPHNs (F4)

*B. TEM:* The TEM image indicates that nanoparticles of optimized felodipine LPHNs (F4) are rounded in shape with nanosize morphology that ascertains the colloidal feature of the F4 as shown in Figure 7.

## Conclusion

 The felodipine LPHNs (H1-H35) that prepared successfully show excellent physical stability according to

 thermodynamic stability evaluation. The newly microwave-based method was used successfully to prepare felodipine LPHNs (H1-H35) that makes it the most advanced method for the preparation of nanocarriers.

 All the optimized felodipine LPHNs formulations (F1-F9) show an extended drug release suitable for control therapeutic agent delivery to improve the patient’s commitment to taking treatment on time. The most optimized formula (F4) shows excellent physicochemical attributes particularly lower globule size and lower PDI in comparison to other felodipine LPHNs (F1-F9) that make it suitable and a new nanosystem for the delivery of both hydrophilic and hydrophobic therapeutic agents.

## Ethical Issues

 All experiments achieved in the present work were approved by a committee supervising scientific research and animal ethics in the university of Baghdad, college of pharmacy for the year 2020/2021.

## Conflict of Interest

 The authors declare there is no conflict of interest.
